# Supplementary material for: Enrichment and sensing tumor cells by embedded immunomodulatory DNA hydrogel to inhibit postoperative tumor recurrence
Source: Nat Commun. 2023 Jul 27;14:4511. doi: 10.1038/s41467-023-40085-4 (PMC10374534; doi:10.1038/s41467-023-40085-4)
Supplement: Supplementary file 1 — Supplementary Information [file 41467_2023_40085_MOESM1_ESM.pdf]

Supplementary Information

Enrichment and sensing tumor cells by embedded immunomodulatory DNA hydrogel to inhibit postoperative tumor recurrence

Danyu Wang [1‡], Jingwen Liu [1‡], Jie Duan [1], Hua Yi [1], Junjie Liu [1,2,3], Haiwei Song [1,5], Zhenzhong Zhang [1,2,3,4]*, Jinjin Shi [1,2,3]*, Kaixiang Zhang [1,2,3]**

[1] School of Pharmaceutical Sciences, Zhengzhou University, Zhengzhou 450001, China

[2] Key Laboratory of Targeting Therapy and Diagnosis for Critical Diseases, Henan Province, China

[3] Key Laboratory of Advanced Drug Preparation Technologies, Ministry of Education, Zhengzhou 450001, China

[4] State Key Laboratory of Esophageal Cancer Prevention & Treatment, Zhengzhou 450001, China

[5] Institute of Molecular and Cell Biology, Agency for Science, Technology, and Research (A*STAR), Singapore 138673, Singapore

*E-mail: zhangkx@zzu.edu.cn; shijinyxy@zzu.edu.cn;

zhangzhenzhong@zzu.edu.cn; haiwei@imcb.a-star.edu.sg

Supplementary Table S1. Sequences used to synthesize DNA hydrogels.

Name	5'-3'
CPDH Template 1	TTACGGGCAGTGGACGCATTGTCTATCAATGAGTT GATGTGGCCCGTTTTTAACGTCAGGAACGTCATGG ATT
CPDH Primer 1	GCGTCCACTGCCCGTAAAATCCAT
CPDH Template 2	AATCCATGTCTCCTTACTTCTATTTTTACGCGCATGT GTGAACTCCGTATGATTGAGCGTCCACTGCCCGTA A
CDH Template 1	TTATGTGGAATTGACACTTGGACCAGCAAGGGGCT TACGTGCCGCCTTTTTAACGTCAGGAACGTCATGG ATT
CDH Primer 1	GTGTCAATTCCACATAAAATCCAT
CDH Template 2	AATCCATGTCTCCTTACTTCTATTTTTACCCTCATCT GTCAACTCCGTATGATTGTGTGTCAATTGCACATAA
CDH Primer 2	ATGGTTTTTATGTGCAATTGACAC
CPDH Primer 2	ATGGATTTTACGGGCAGTG GACGC
DH Template 1	TTATGTGGAATTGACACTTGGACCAGCAAGGGGCT TACGTGCCGCCTTTTTTTCGACAGCACACACTAGC ATT
DH Primer 1	GTGTCAATTCCACATAAAATGCTA
DH Template 2	AATGCTAGAGACCTTACATCTATTTTTACCCTCATCT GTCAACTCCGTATGATTGAGTGTCAATTGCACATAA
DH Primer 2	TAGCATTTTATGTGCAATTGACAC
CpG cDNA-Ce6	Ce6-AACGTCAGGAACGTCATGGA
rCpG cDNA-Ce6	Ce6-TTCGACAGCACACACTAGCA

Supplementary Table S2. Oligonucleotide sequences used to synthesize ATP sensors.

Name	5'-3'
L1-ATP Aptamer-BHQ2	BHQ2- CAGTCACCTGGGGGAGTATTGCGGAGGAAGGTTTTTG TCACCGAAAAAGTCACCACCTT
P1	GAGTGCAGGCGACGGC
L2-cApt	GCCGTTTTACTCCGTATGATTGAGAAAACCAGGTGACT G* Cy5CGATTGGTGACAAAAAGTGG
L3-rATP Aptamer-BHQ2	BHQ2- CAGTCACCTGGGGGAGTATTGCAAAAACAGGTTTTTG TCACCGAAAAAGTCACCACCTT
Control-L1	BHQ2-CAGTCACCTGGGGGAGTATTGCGGAGGAAGGT
Control-L2	ACTCCGTATGATTGAGAAAACCAGGTGACTG-Cy5

Supplementary Table S3. Sequences for analysis of CpG and PDL1.

Name	5'-3'
AptPDL1/MB-FAM	FAM-ACGGGCAGTGGACGCATTGTCTATCA
AptPDL1/MB-BHQ	TGCGTCCACTGCCCGT-BHQ
CpG/MB-FAM	FAM–CGTTAACGTCAGGAACGTCATGGATTCC
CpG/MB-BHQ	CCTGACGTTAACG-BHQ
Genetic analysis sequences	ACTGCCCGTAAAATCCATGACGTTCCTGACGTTAAAA ACGGGCCACATCAACTCATTGATAGACAATGCGTCCA CTGCCCGTAAAATCCATGACGTTCCTG
Primer 1	AATCCATGACGTTCCTGAC
Primer 2	TGGATTTTACGGGCAGTGGA

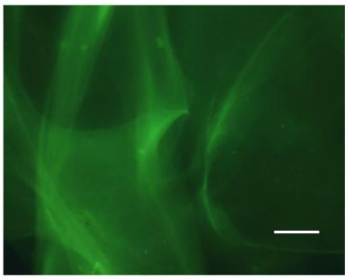

Supplementary Figure 1. Fluorescence microscopy image of CPDH-Ce6 (stained with SYBR Green II). Scale bar: 50 μm. The experiments were repeated three times independently.

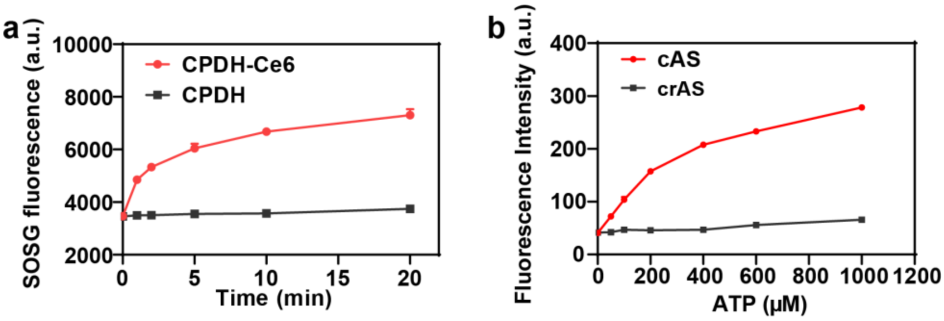

Supplementary Figure S2. a) $^1O_2$ produced by photoactivated CPDH-Ce6 and CPDH at different times (n = 3). b) Fluorescence intensity of cAS and crAS in response to different concentrations of ATP (n = 3). Data are presented as mean ± SD. Source data from (Figure S2) are provided as a Source Data file.

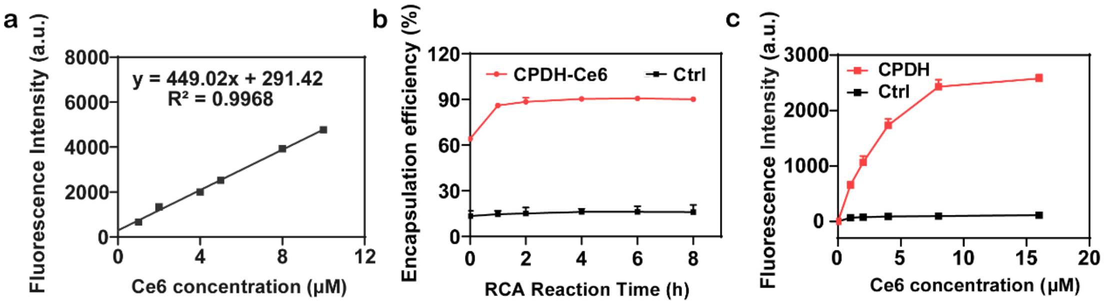

Supplementary Figure S3. a) Standard curve of Ce6. b) Encapsulation rate of Ce6 added during RCA reaction (n = 3). c) Analysis of Ce6 loading capacity of CPDH by fluorescence intensity. Data are presented as mean ± SD. Source data from (Figure S3) are provided as a Source Data file.

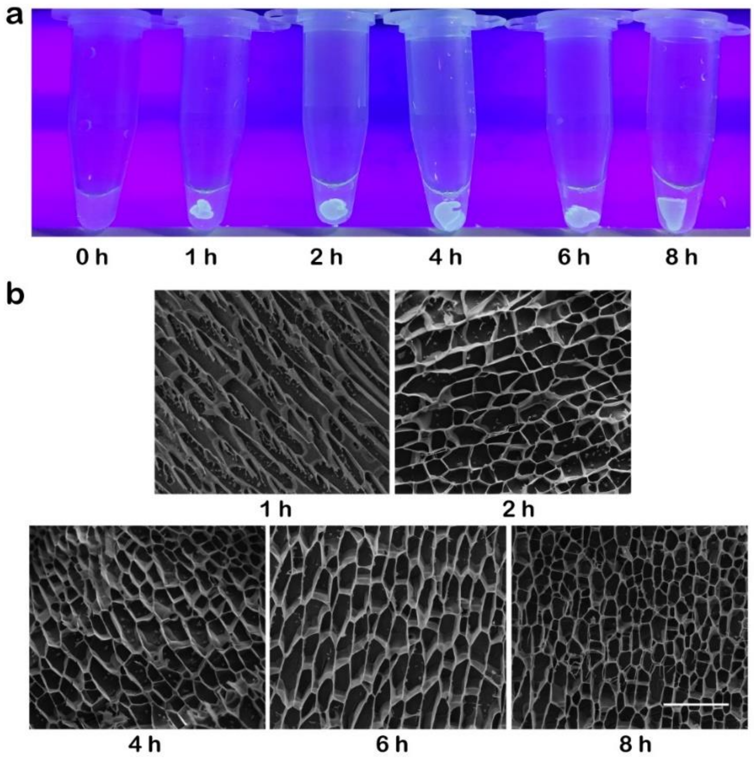

Supplementary Figure S4. The DNA hydrogels synthesized by adding Ce6 at different times of the RCA reaction were visualized in pictures a) and cryo-SEM b). Scale bar: 50 μm.

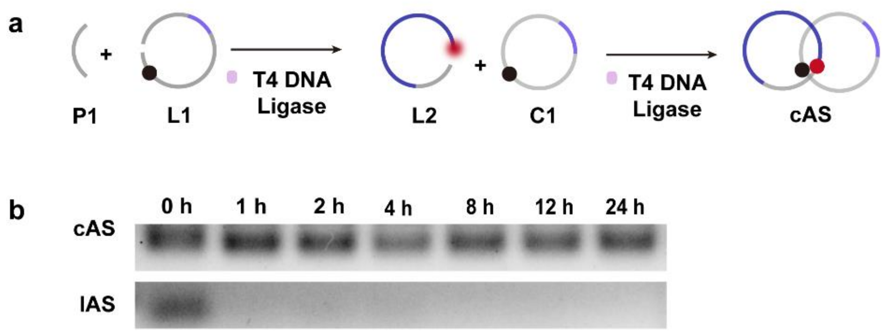

Supplementary Figure S5. a) Schematic diagram of circular ATP sensor synthesis. b) 2% agarose gel analysis of circular ATP sensor and linear ATP sensors after treatment with 0.15 U/ μL Exo I and 0.5 U/ μL Exo III for different time spans.

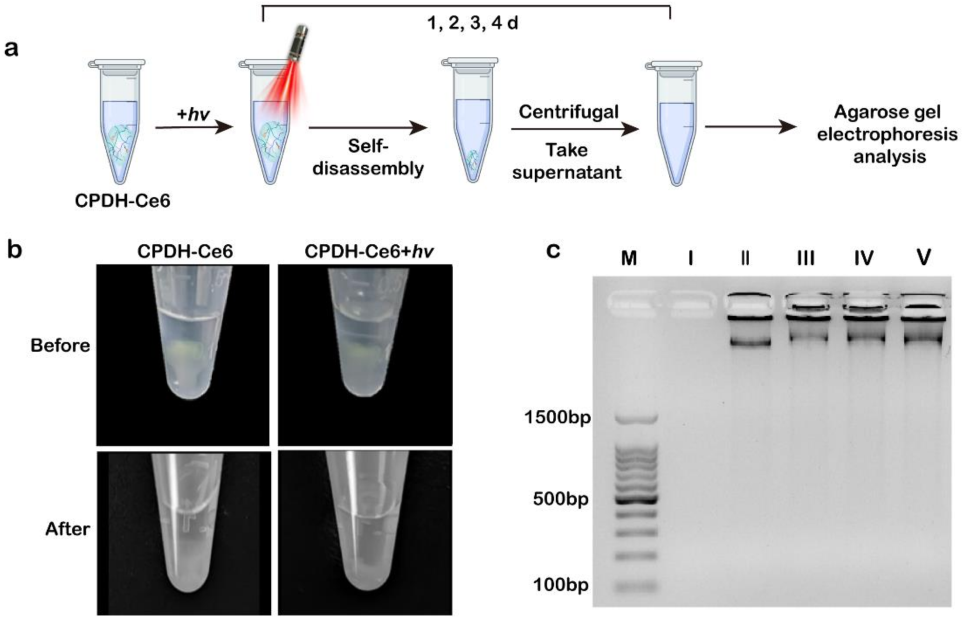

Supplementary Figure S6. a) Schematic diagram for the analysis of DNA fragments released from the CPDH-Ce6 irradiated by a 660 nm (0.2 W cm^{-2}) laser. b) Visualization of morphological changes of CPDH-Ce6 before and after incubation at 37°C for 5 days (with or without 10 min laser irradiation). c) The supernatants were analyzed by agarose gel electrophoresis for the degradation of CPDH-Ce6 treated with different conditions. M: DNA ladder, I: no irradiation. II: incubation for 1 d after irradiation, III: incubation for 2 d after irradiation, IV: incubation for 3 d after irradiation, V: incubation for 4 d after irradiation.

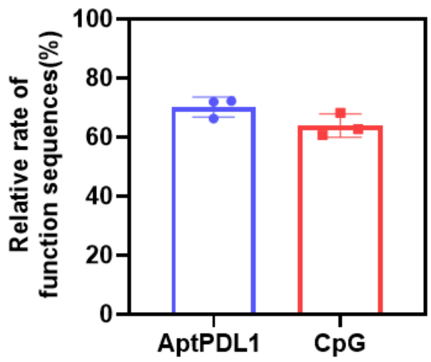

Supplementary Figure S7. Characterization of the release rates of functional PDL1 aptamers and CpG sequences released from laser-irradiated CPDH-Ce6 (n = 3). Data are presented as mean ± SD. Source data from (Figure S7) are provided as a Source Data file.

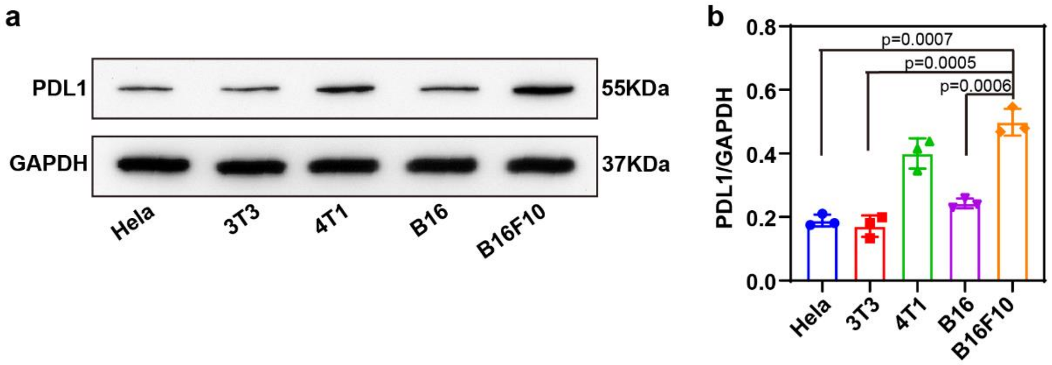

Supplementary Figure S8. The expression levels of PDL1 protein in different tumor cells (Hela, 3T3, 4T1, B16 and B16F10) were analyzed by a) western-blot and b) semi-quantification with Image-J software (n = 3). Data are analyzed by two-sided Student's *t*-test and shown as mean ± SD. Source data from (Figure S8b) are provided as a Source Data file.

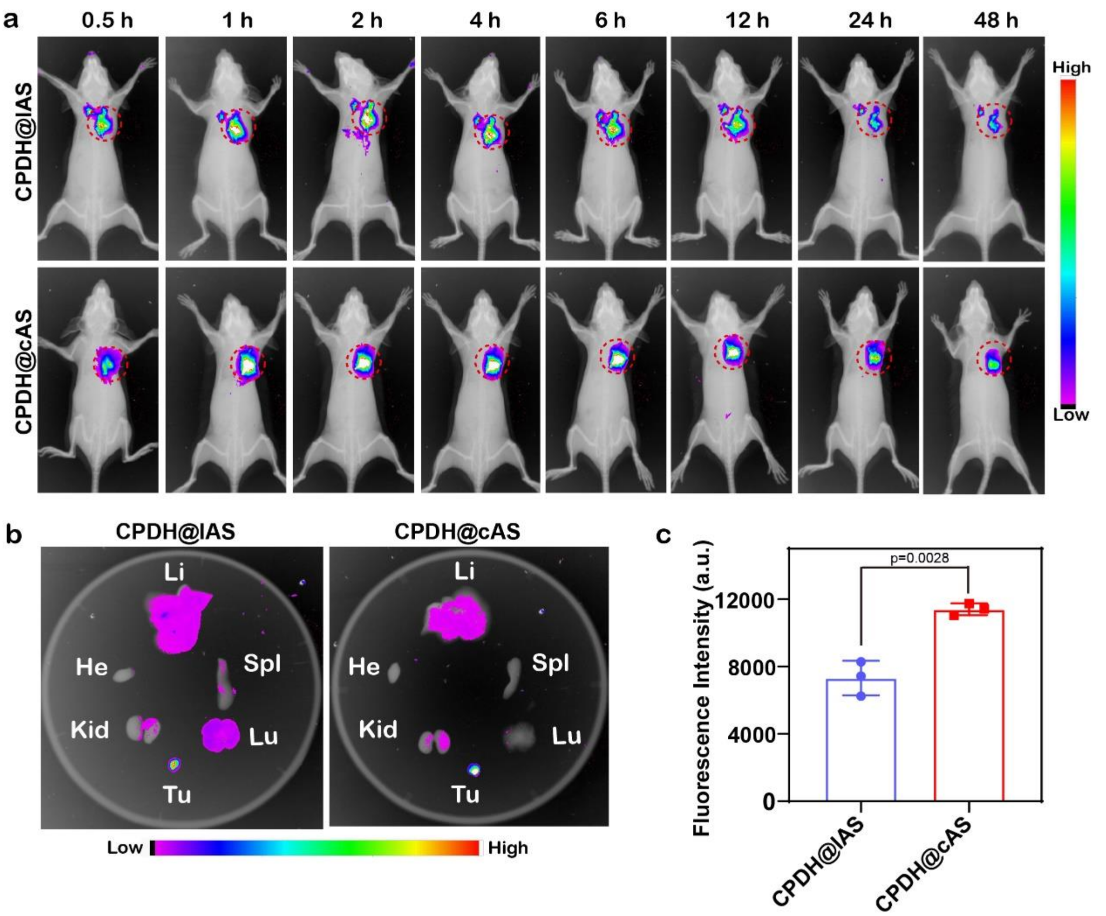

Supplementary Figure S9. a) *In vivo* fluorescence images of mice with tumor recurrence after embedding different hydrogel sensors. b) Fluorescence images of isolated tumors and organs (heart, liver, spleen, lung, kidney) harvested 48 h after embedding. c) Cy5 fluorescence intensity of circular ATP sensor and linear ATP sensor in tumor region at different imaging times (n = 3). Data are analyzed by two-sided Student's *t*-test and shown as mean ± SD. Source data from (Figure S9c) are provided as a Source Data file.

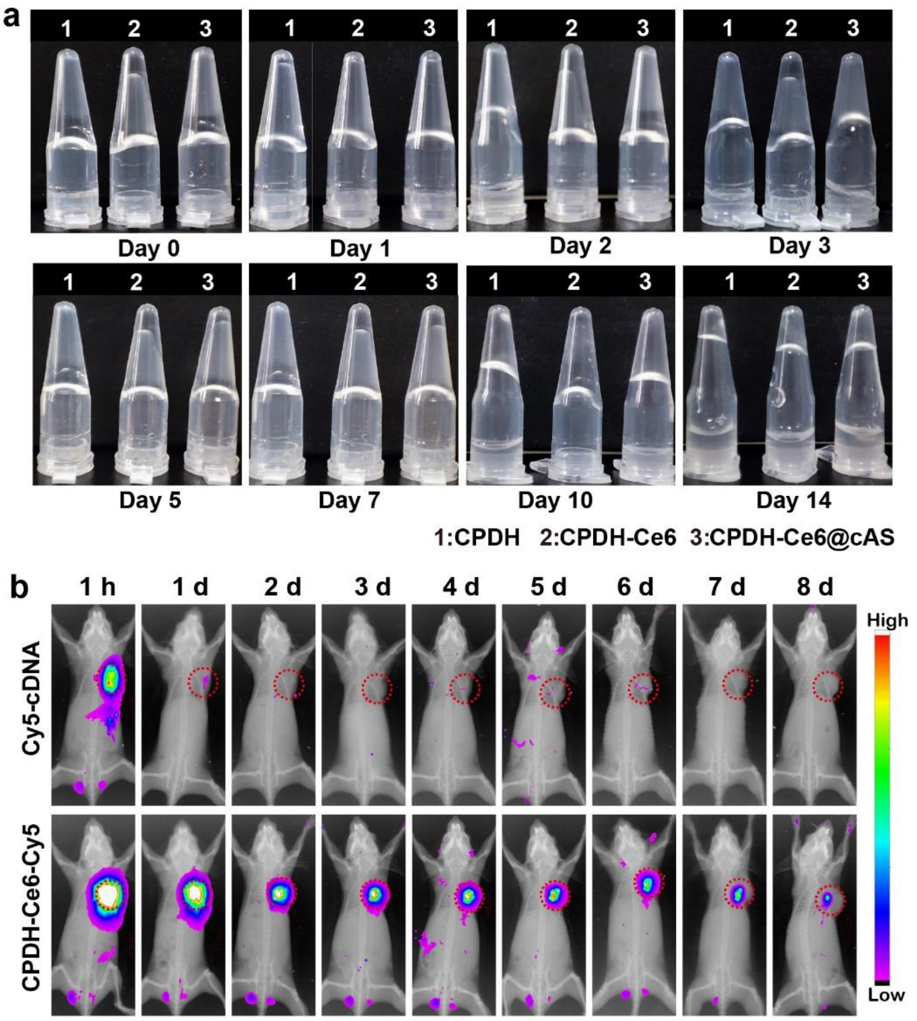

Supplementary Figure S10. Evaluation of CPDH-Ce6 stability *in vitro* and *in vivo*. a) Morphological visualization of DNA hydrogels stored in 10% FBS for 14 days. b) In vivo imaging characterizing the *in vivo* retention of Cy5 fluorescence in Cy5-cDNA and CPDH-Ce6-Cy5 encapsulated at the surgical site in mice.

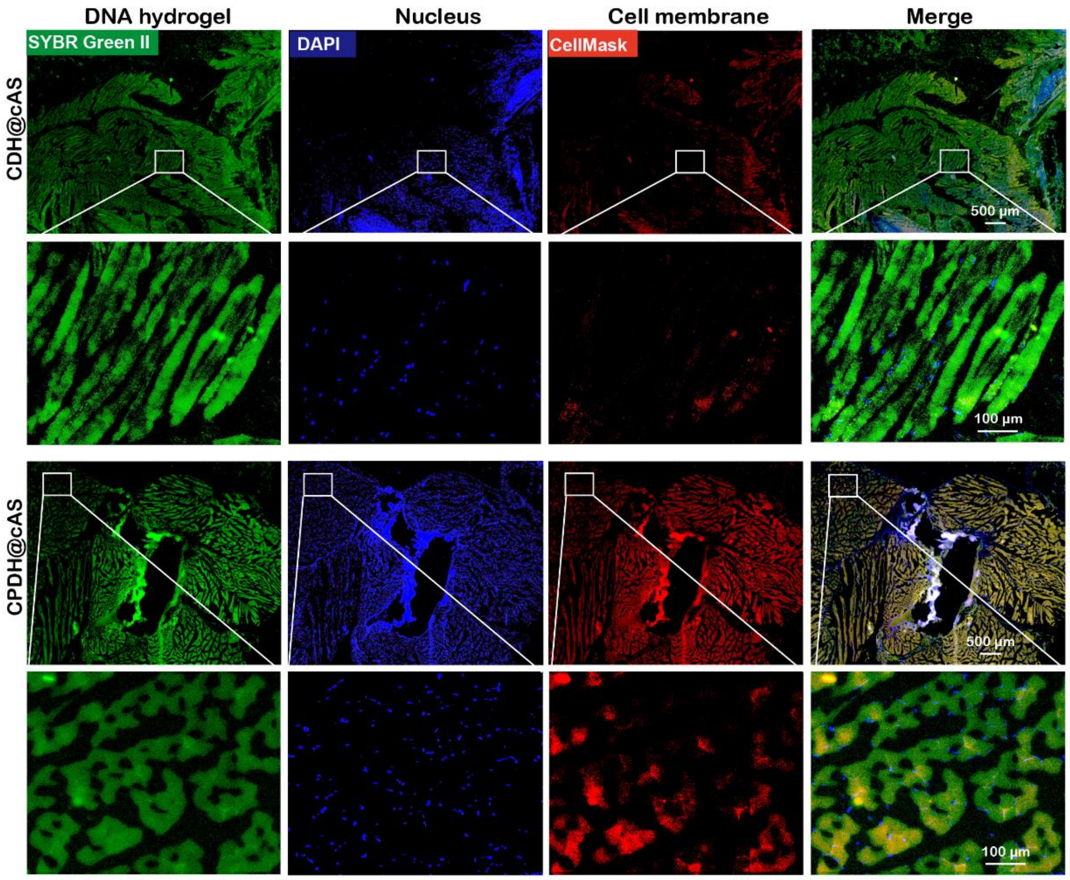

Supplementary Figure S11. Frozen section fluorescence imaging of DNA hydrogels (with or without PDL1 aptamer) capturing tumor cells *in vivo* and local magnification images. Green: DNA hydrogel, Blue: cell nucleus, Red: tumor cell membrane. Scale bar: 500 μm (top) and 100 μm (bottom). The experiments were repeated three times independently.

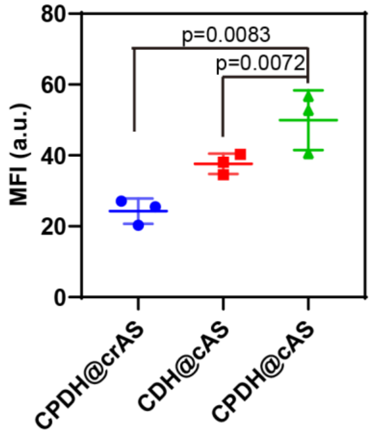

Supplementary Figure S12. Mean fluorescence intensity of ATP sensors from tumor sections of different treated mice (n = 3). Data are analyzed by two-sided Student's *t*-test and shown as mean ± SD. Source data from (Figure S12) are provided as a Source Data file.

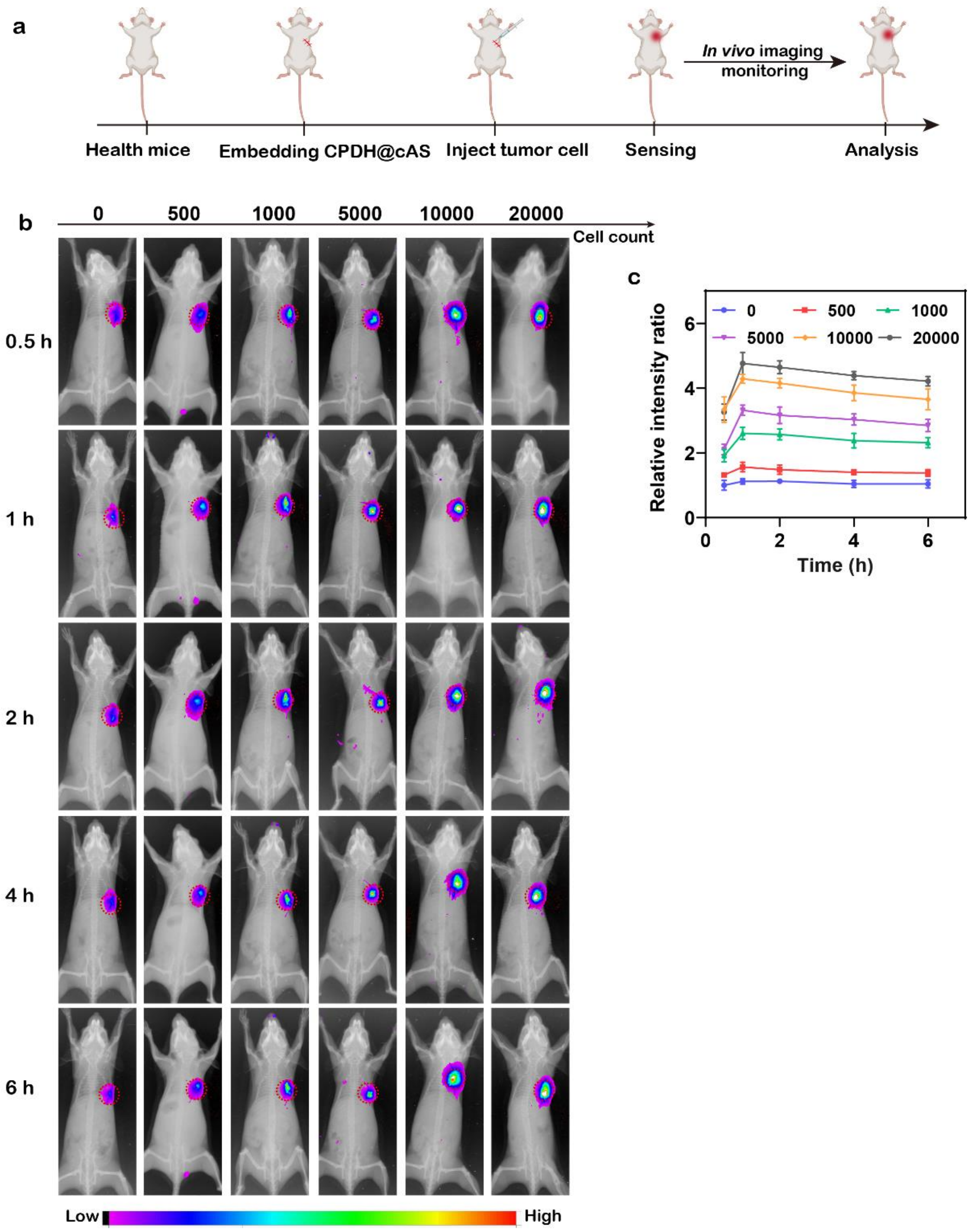

Supplementary Figure S13. a) Schematic diagram of detecting tumor recurrence *in vivo*. b) Detection of different numbers of tumor cells by fluorescence imaging *in vivo* after encapsulating CPDH@cAS. c) Relative fluorescence quantification of corresponding fluorescence images in b). Data are presented as mean ± SD (n = 3). Source data from (Figure S13c) are provided as a Source Data file.

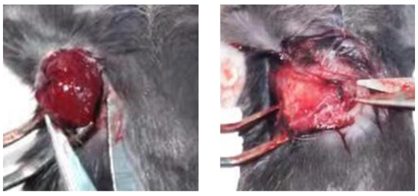

Supplementary Figure S14. Magnified photos of the tumor area before and after surgery.

Supplementary Table S4. Primary and distal tumor inhibition rates (TIR) were calculated for different groups of mice

**Primary tumor**	**Average bioluminescence intensity**	**TIR**
Untreated	14.67×10^5 (BI $_{T0}$)	0%
CPDH-Ce6	11.16×10^5 (BI $_{T1}$)	23.9%
DH-Ce6+*hv*	5.184×10^5 (BI $_{T2}$)	64.7%
CPDH-Ce6+*hv*	1.7416×10^5 (BI $_{T3}$)	88.1%
**Distant tumor**	**Average bioluminescence intensity**	**TIR**
Untreated	13.34×10^5 (BI $_{T0}$)	0%
CPDH-Ce6	12.174×10^5 (BI $_{T1}$)	8.7%
DH-Ce6+*hv*	7.246×10^5 (BI $_{T2}$)	45.7%
CPDH-Ce6+*hv*	5.83×10^5 (BI $_{T3}$)	56.3%

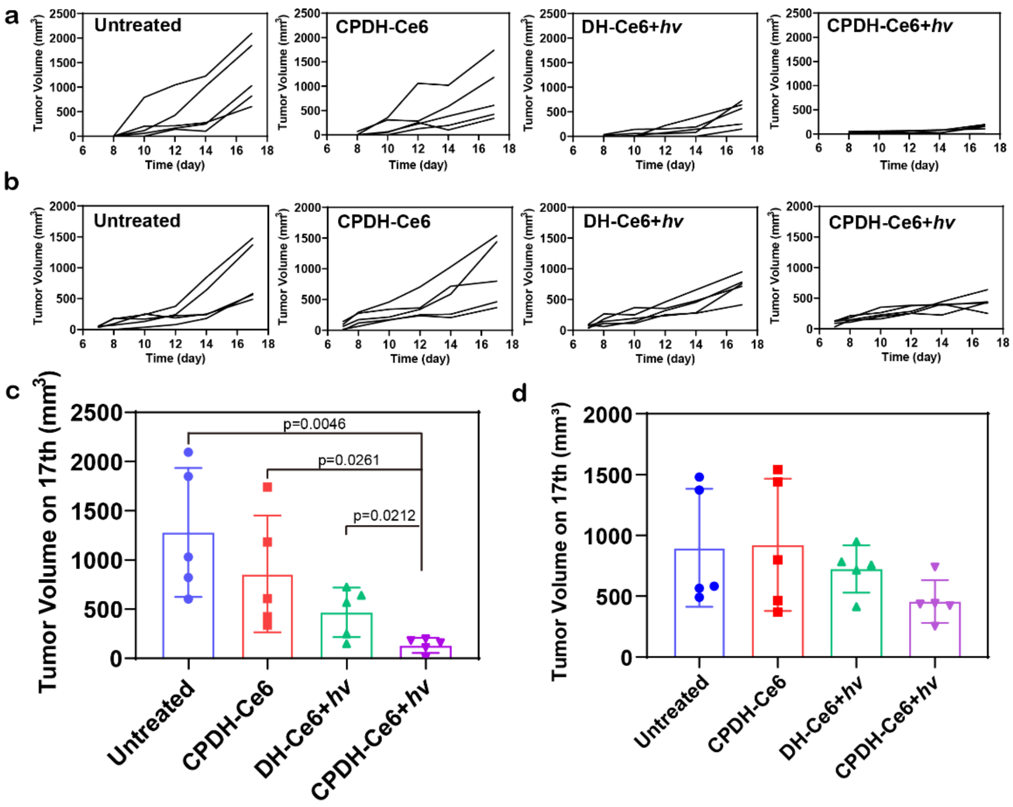

Supplementary Figure S15. a) Individual recurrent and b) the corresponding individual distant tumor growth curves of mice after different treatments. Comparison of c) recurrent and d) distant tumor volume on 17th after treatment. Laser irradiation (+) was performed with a 660 nm laser (0.2 Wcm^{-2}, 10 min). Data are analyzed by two-sided Student's *t*-test and shown as mean ± SD (n = 5). Source data from (Figure S15) are provided as a Source Data file.

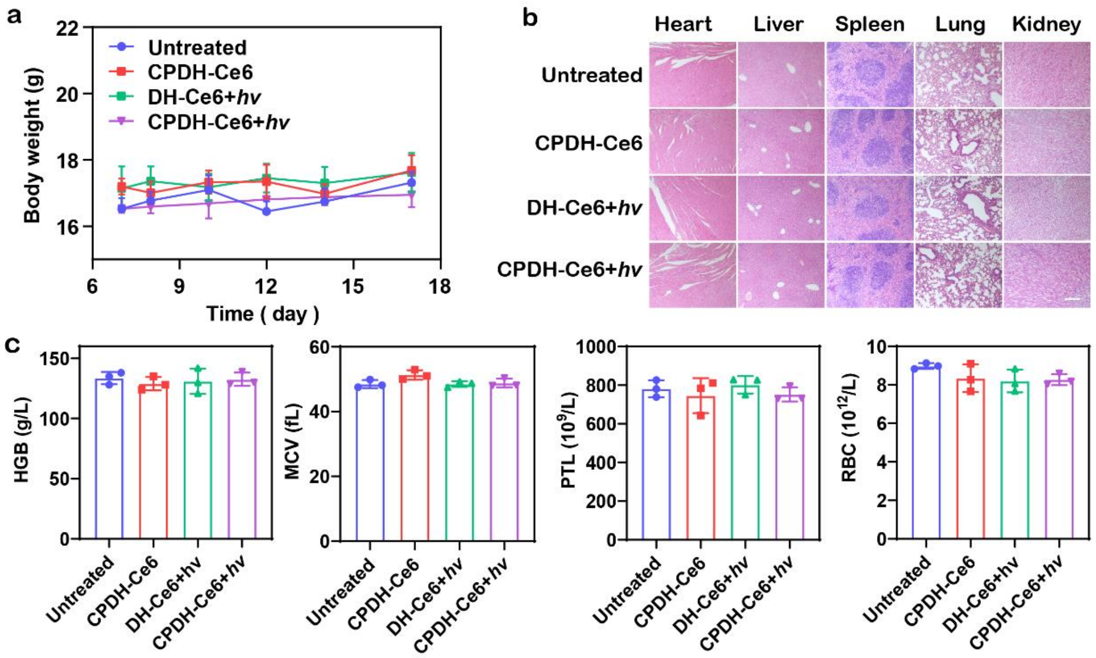

Supplementary Figure S16. Biosafety analysis of CPDH-Ce6. a) Body weight curve of mice after different treatments (n = 5). b) H&E stained micrographs of major organs of mice collected on day 17 from untreated, CPDH-Ce6, DH-Ce6+*hv,* and CPDH-Ce6+*hv*. Scale bar: 200 μm. c) Blood hematology analysis of the mice after administration for 17 days (n = 3). Data are presented mean ± SD. Source data from (Figure S16a and S16c) are provided as a Source Data file.

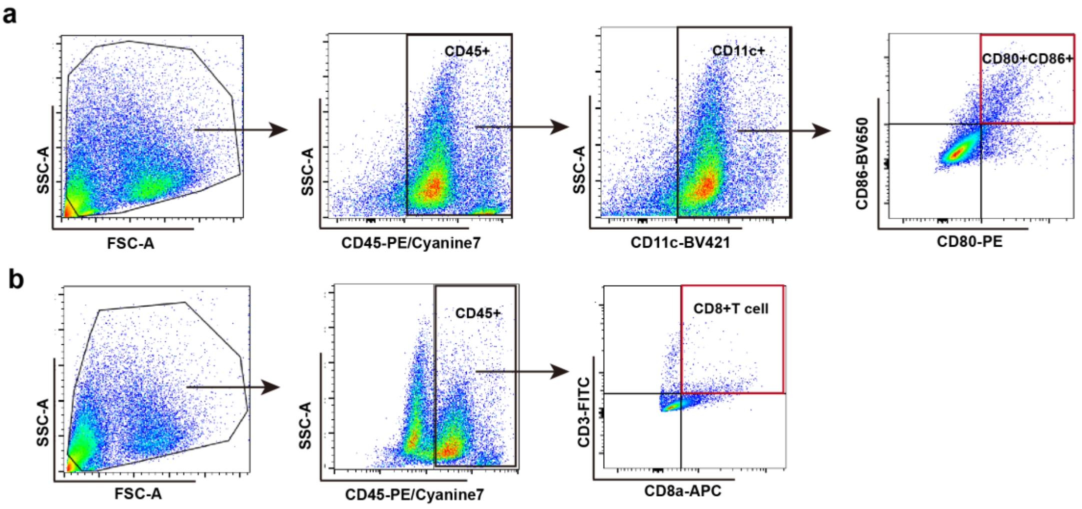

Supplementary Figure S17. Gating strategy for analyzing immune cell population in tumor tissues. Gating strategies to analyze a) $CD45^+$ $CD3^+$ $CD8^+$, b) $CD11c^+$ $CD80^+$ $CD86^+$.

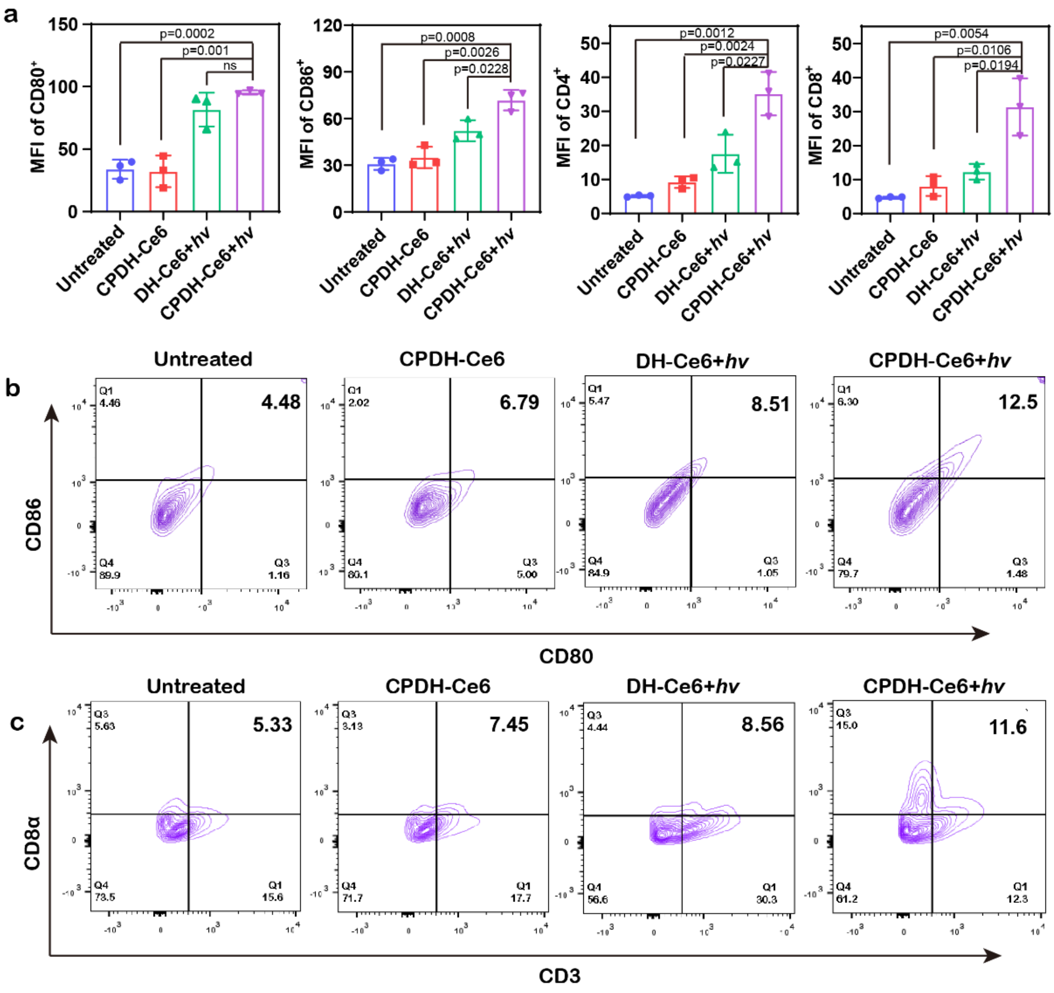

Supplementary Figure S18. a) The corresponding quantified of MFI of CD80$^+$, CD86$^+$, CD4$^+$ and CD8$^+$ in primary tumors (n = 3). Data are analyzed by two-sided Student's *t*-test and shown as mean ± SD. b) Flow cytometry analysis of distant tumor-infiltrating DCs (CD80$^+$CD86$^+$) and c) CTLs (CD3$^+$CD8α$^+$) after various treatments. Source data from (Figure S18a) are provided as a Source Data file.

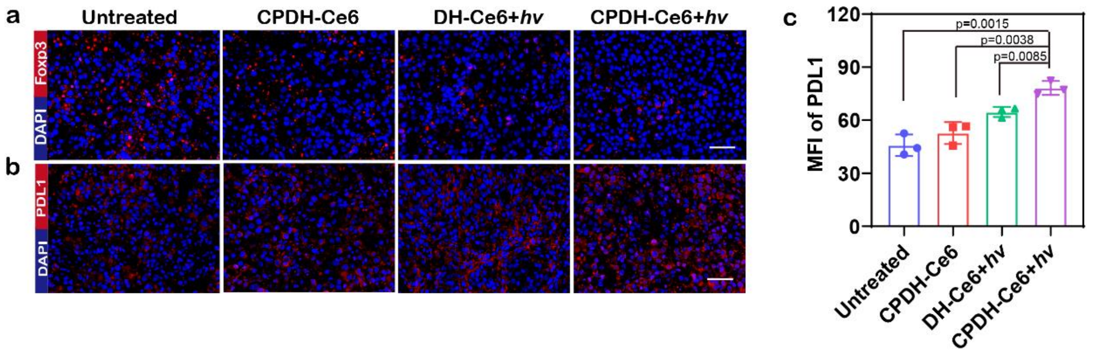

Supplementary Figure S19. a) Immunofluorescence staining of Foxp3-labeled Treg cells and b) PDL1 in B16F10 tumor tissue after different treatments. Scale bars: 50 µm. c) The corresponding quantification of PDL1 mean fluorescence intensity (n = 3). Data are analyzed by two-sided Student's *t*-test and shown as mean ± SD. Source data from (Figure S19c) are provided as a Source Data file.

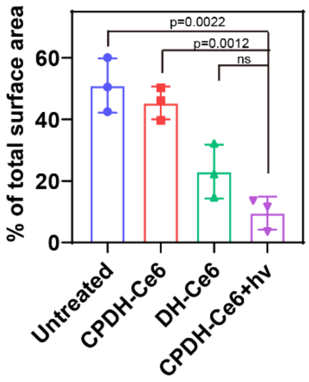

Supplementary Figure S20. Quantification of the percentage of lung area occupied by metastases in H&E-stained sections (n = 3). Data are analyzed by two-sided Student's *t*-test and shown as mean ± SD. Source data from (Figure S20) are provided as a Source Data file.

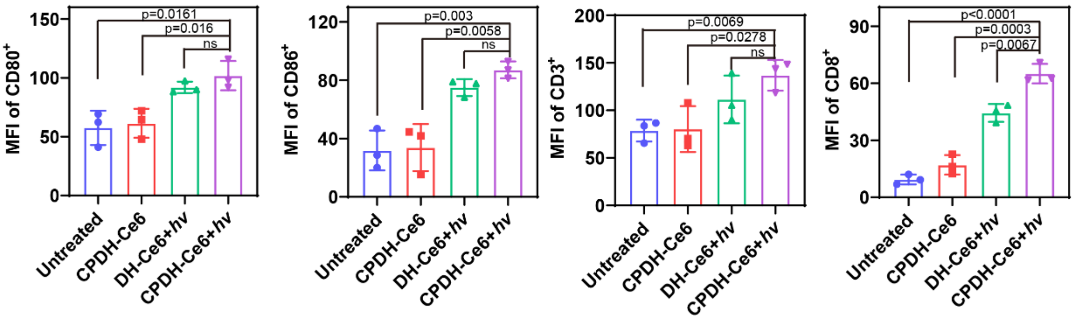

Supplementary Figure S21. Analysis of fluorescence intensity of $CD80^+$ and $CD86^+$ DC cells, $CD3^+$ and $CD8^+$ T cells in lung sections (n = 3). Data are analyzed by two-sided Student's *t*-test and shown as mean ± SD. Source data from (Figure S21) are provided as a Source Data file.
